# Web review: Anatomy atlases

**Published:** 2008-05

**Authors:** Cdr IK Indrajit Surg

**Affiliations:** Department of Radiodiagnosis and Imaging, Army Hospital (Research and Referral), Delhi Cantt - 110 010, India

Few web-based anatomy atlases related to radiology and imaging that are available at the moment are reviewed below:

1. **Anatomy Atlases** at http://www.anatomyatlases.org is a digital library of anatomy information curated by Ronald A. Bergman, Ph.D. This website links to a set of useful educational anatomy atlases that comprise the following: Atlas of Human Anatomy, Atlas of Human Anatomy in Cross Section, Atlas of Microscopic Anatomy - a Functional Approach, Illustrated Encyclopedia of Human Anatomic Variation, and Lessons from a Bone Box.
2. **Compare** is a website created and authored by M. Grunewald, Department of Diagnostic Radiology, University of Erlangen-Nuremberg, Germany; it is available at http://www.idr.med.uni-erlangen.de/COMPARE/Ecomparetitlepage.htm. Various plain film images of the thorax and abdomen; CT scan images of the brain, thorax, abdomen, and pelvis; and angiogram images of the carotid, pulmonary, abdominal, mesenteric, renal, and the upper and lower limb arteries, including the aortic arch, are available. Normal and pathological images of water-soluble contrast/barium swallow, double-contrast barium meal, small-bowel enema, hysterosalpingography, micturating cystourethrography, and urography are also featured.
3. **Radiologic Anatomy Browser** is sourced from the Uniformed Services University and is accessible at http://rad.usuhs.mil/rad/iong/index.html. The Anatomy Browser in an outline format permits expanding or collapsing levels. The material produced by S. Rudinsky and J. G. Smirniotopoulos is catalogued into regions such as Head and Neck, Back and Upper Extremity, Chest, Abdomen, and Pelvis, etc. The topics are comprehensively covered and a checklist is an added bonus at the end of the sections.
4. **Normal Radiologic Anatomy** is a basic primer on Anatomy designed for the Radiologist; it is available at http://lib.cpums.edu.cn/jiepou/tupu/atlas/www.vh.org/adult/provider/radiology/NormalRadAnatomy/index.html. This site is authored by a team headed by J. Livermore and W. Erkonen and the online atlas covers x-ray, CT, MRI, and ultrasound. In essence, the image material offers information on anatomical areas such as the head and neck, thorax, abdomen, pelvis, and the upper and lower extremity. A special section on 3D reconstructions is also available.
5. **Radiology Anatomy** is accessible at http://sprojects.mmip.mcgill.ca/radiology/. The material covers regions including the thorax, pelvis, abdomen, spine, extremities, and the head and neck. Each region has a varied set of modalities describing the regional structures. Besides this, there are online quizzes to reinforce learning of various anatomic structures.
6. **Radiology Anatomy Models**, from University of Washington, is a set of radiology and anatomy teaching files, available at http://www.rad.washington.edu/anatomy/index.html. The material covers areas such as normal knee anatomy, normal distal thigh anatomy, radiographic anatomy of the upper and lower extremity muscles, etc. Added features in this atlas include the radiographic evaluation of hallux valgus, ultrasound of the shoulder, and a TMJ tutorial, all of which are very well illustrated.
7. **Radiologic Anatomy** is a website dealing with basic gross anatomy in radiographic studies. Developed and sourced from the Wayne State University School of Medicine and available at http://www.med.wayne.edu/diagRadiology/Anatomy_Modules/Page1.html, it has distinct sections dealing with the brain, upper abdomen, thorax, pelvis, upper thigh, and skull base.
8. **Digital Anatomist Project** is available from University of Washington at http://www9.biostr.washington.edu/da.html. The site is famous for hosting its interactive atlases on the brain, knee, and the thoracic viscera in a fascinating and futuristic style. In particular, ‘The Thoracic Viscera’ by D. M. Conley and C. Rosse is an excellent area. Derived from cadaver sections, MRI scans, and computer reconstructions, there are illustrative features on 2D and 3D views of the brain, cortical connections, 3D views of thoracic organs, and 2D and 3D views of the knee.
9. The **Anatomy Wiz!** is an educational and reference tool, available at http://www.bridgeporthospital.org/GME/anatomywiz/awiz.aspx. Designed and authored by Gerard J. Muro, M.D, it has multiple modules focused on ENT and musculoskeletal anatomy, wherein structures, discussion, and references are offered for various structures on cross-sectional images. Currently available anatomical topics include brain stem anatomy, CT angiography, anatomy of the abdomen and pelvis, MRI anatomy of the elbow, neck anatomy, temporal bone anatomy on multi-detector CT (MDCT), orbit anatomy, and skull base and soft tissue neck anatomy on MDCT.
10. **Free Interactive Atlas of Human Anatomy** is available at http://www.e-anatomy.org/. ‘A user-friendly interface allows to cine through multi-slice image series combined with interactive textual information, 3D models, and anatomy drawings. More than 1500 slices from normal CT and MR exams were selected in order to cover the entire sectional anatomy of human body.’ Designed and hosted by Campus Medica, the site is a RSNA 2007 award winner ‘for excellence in the design, development, and implementation of e-Anatomy.’

## End piece

**LUMEN Cross-Section Tutorial** is authored by Dr. John A. McNulty and offers cross-sectional images from the Visible Human Project-NLM. Available at http://www.lumen.luc.edu/lumen/meded/grossanatomy/x_sec/mainx_sec.htm, the material features sections on the head and neck, thorax, abdomen, limbs, and pelvis.

The **Whole Brain Atlas** at http://www.med.harvard.edu/AANLIB/home.html is a project maintained by Keith A. Johnson and J. Alex Becker, from the Departments of Radiology and Neurology, at Brigham and Women's Hospital, Harvard Medical School. Within the atlas, useful sections available include a Neuroimaging Primer, Vascular Anatomy, Normal Anatomy in 3D with MRI/PET, and a section on cerebrovascular, neoplastic, degenerative, inflammatory, and infectious diseases.

**Thoracic Anatomy** at http://www.chestx-ray.com/Anatomy/Anatomy.html is hyperlinked to five radiology and imaging anatomy sections, each rich in graphics: Cross-sectional Anatomy, Mediastinal Anatomy, Inflated Lungs - Virtual Reality, Segmental Projections, and Introduction to Cardiothoracic Imaging.

**CTisUS** at http://www.ctisus.org/ is an outstanding portal administered by E. K. Fishman, MD, and sourced from the Department of Radiology, Johns Hopkins University. Maintained by the Advanced Medical Imaging Laboratory (AMIL). The site makes learning cardiac CT meaningful, with classification of the material into four key areas: Most Popular, Scanner Protocols, Learning Modules, and Everything Else.

Within these, there are sections on 64-slice MDCT, anatomy organ systems, CT angiography, and CT/PET. The Scanner Protocols section has technical material on almost all types of scanners: single detector, 4-slice, 16-slice, and 64-slice, including Siemens Definition protocols. For the students, Teaching Files, Journal Club, and Quiz of the Month may be particularly useful. The teaching file modules are available at http://www.ctisus.org/tf/index.html and feature illustrative cases related to various body parts.

Two other interesting sections are Ask the Fish CT and Imaging Pearls. The Pearls Archive at http://www.ctisus.org/pearls/index.html has useful conclusive statements on various regions as well as Oral Contrast, IV Contrast, Radiation Dose, Screening CT, Technology, Virtual Imaging, and 3D and Workflow. The podcasting and vodcasting sections are available at http://www.ctisus.org/rsna_2006/podcasting/ and http://www.ctisus.com/vodcasts/index.html.

**Pedradinfo** at http://www.pedrad.info/is a ‘peer-reviewed, pediatric radiology platform on the Web’ maintained by Roland Talanow. The website which is an interactive radiology teaching platform has been given awards at the RSNA 2005 and ARRS 2006. It allows online book searches at http://www.kinderradiologie-online.de/radiology/Search.shtml/or browsing of interesting cases at http://www.kinderradiologie-online.de/radiology/Most_Interesting_Cases.shtml.

